# Pre-operative Traction in Severe Rigid Kyphoscoliosis - CT-based Navigation Pelvic Pin Insertion in Halo-Pelvic Traction: A Case Report

**DOI:** 10.5704/MOJ.2503.014

**Published:** 2025-03

**Authors:** KS Tan, P Devarani, C Saturveithan, CYW Chan, A Saw

**Affiliations:** National Orthopaedic Centre of Excellence for Research and Learning (NOCERAL), Department of Orthopaedic Surgery, Universiti Malaya, Kuala Lumpur, Malaysia

**Keywords:** severe rigid kyphoscoliosis, pre-operative traction, halo-pelvic traction, CT-based navigation pelvic pin insertion, turner syndrome

## Abstract

Neglected severe rigid kyphoscoliosis can lead to rapid curve progression, presenting a challenge for surgical correction and carrying higher risks of mortality, morbidity, and neurological injury, potentially resulting in permanent paralysis. Halo-pelvic traction (HPT) has been reported to be effective in improving curve flexibility, assisting the surgical correction process, and reducing the likelihood of neurological complications. We report the case of a 15-year-old girl with mosaic Turner syndrome and severe kyphoscoliosis, who experienced progressive curve progression (from 41° to 158°) over a span of 6 years. Preoperative halo gravity traction (HGT) was unsuccessful. To address this deformity, HPT was performed with CT-based navigation for pelvic pin insertion, considering her relatively small pelvis and pelvic obliquity. This technique allowed for precise pin placement, reducing the risk of injury to major arteries, nerves, and abdominal/pelvic organs, while enabling the creation of a more versatile halo-pelvic frame designed to enhance patient comfort and mobility. The patient underwent weekly distraction using HPT for 4 weeks, during which her coronal Cobb angle reduced from 158° to 103° and her kyphotic angle decreased from 90° to 64°. With this notable improvement in the primary spinal curvature, we proceeded with posterior spinal fusion. Notably, this approach obviated the need for vertebral column resection. As a result, we achieved a correction rate of 53.8% in the coronal Cobb angle and 55.6% in the kyphotic angle without neurological injury.

## Introduction

Severe rigid scoliosis is defined when Cobb angle was >90° and flexibility was <30° based on bending films^1^. This deformity can progress rapidly if untreated, leading to complications such as cardiopulmonary impairment and increased mortality rates. Early and aggressive surgical intervention is necessary to prevent further deterioration, but it is challenging and carries significant risks, including substantial bleeding, neurological injury, paralysis, pseudoarthrosis, and implant failure^2,3^. Pre-operative methods like halo-gravity traction (HGT) have limited efficacy in severe cases^3^. Conversely, halo-pelvic traction (HPT) effectively improves curve magnitude and flexibility, facilitating corrective surgery and reducing neurological complications^1^. Mosaic Turner syndrome, a variant where only some cells lack an X chromosome (45, X / 46, XX), presents milder phenotypes compared to classic Turner. Approximately 10% of Turner syndrome patients develop scoliosis, and nearly all exhibit short stature with a small pelvis^4^. Conventional pelvic pin placement technique is risky due to atypical pelvic morphology in syndromic patients, potentially injuring major arteries, nerves, and organs.

We report a case using CT-based navigation for precise pelvic pin insertion to construct HPT for pre-operative distraction of severe rigid kyphoscoliosis which ultimately enabled surgical correction via posterior spinal fusion without requiring vertebral column resection.

## Case Report

**Fig. 1:**
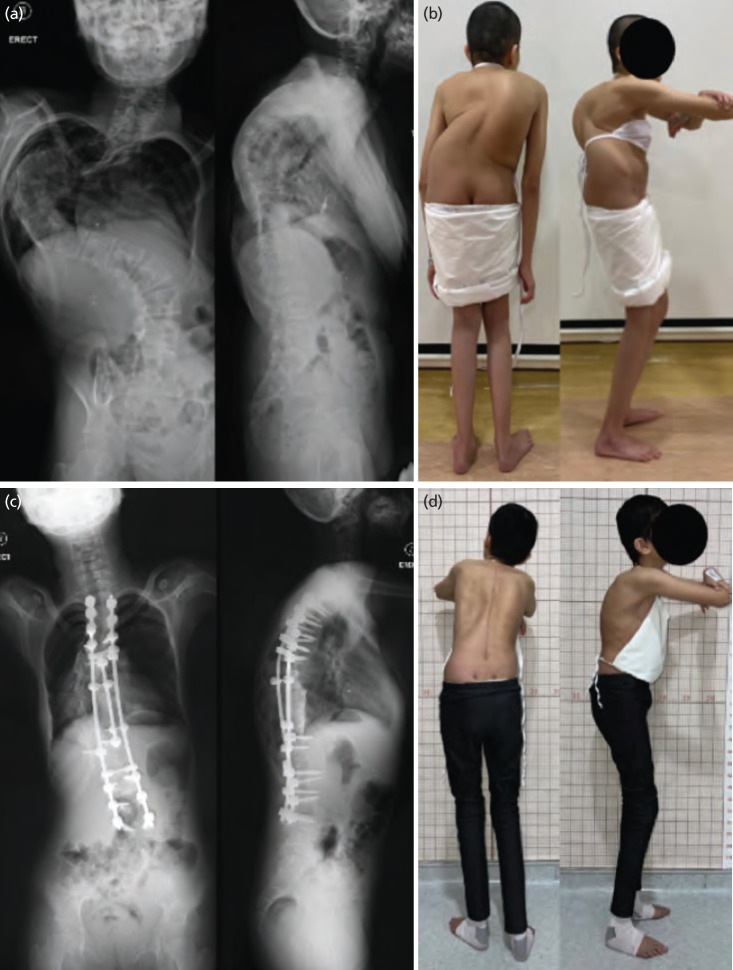
(a) Anterior-posterior and lateral view of the erect spine plain radiograph showing a Cobb angle 158° and kyphotic angle 90°. (b) Clinical photographs of the patient before HGT and HPT in posteroanterior view showed neck tilt, right truncal shift with prominent thoracic hump and uneven shoulder level. The right sagittal view showed thoracic kyphosis malalignment. (c) Anterior-posterior and lateral view of the erect spine plain radiograph after posterior spinal fusion from T1 to L3, showing Cobb angle of 73° and kyphotic angle of 40°. (d) Clinical photographs of patient show improved scoliosis and thoracic kyphosis with height increase from 137cm to 153cm.

**Fig. 2:**
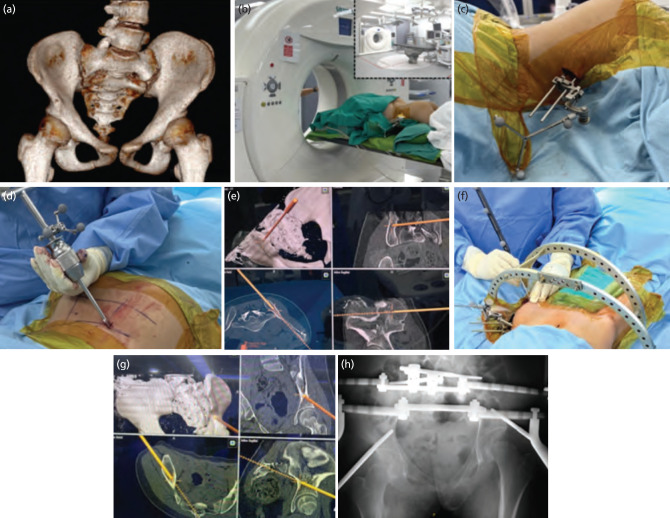
(a) 3D reconstruction CT image of pelvis show pelvic rotation. (b) CT scans were performed in the operating theatre to calibrate the navigation system. (c) Two 3.5mm Schanz pins were inserted into the left iliac crest to secure the navigation tracker. (d) The navigation tracker was attached to the T-handle to guide the insertion of the HA pin. (e) Images showed insertion of the HA pin into the posterior iliac wing under navigation guidance, with a user interface displaying pelvis in 3D, coronal, axial and sagittal views. (f) The navigation probe was used to determine the trajectory and optimal position of the supra-acetabular pin. (g) Images on the user interface show the position of inserted supraacetabular HA-pin. (h) Pelvic radiograph showed final pelvic pin placement in HPT.

A 15-year-old girl with underlying mosaic Turner syndrome, short statue (height 137cm, BMI 16.3 kg/m^2^), congenital hypothyroidism, and a repaired atrial septal defect, presented with kyphoscoliosis deformity since the age 2. Her condition worsened significantly from age 9, with her Cobb angle increasing from 41° to 158° and her kyphotic angle from 51° to 90° by age 15 (Fig. 1a and Fig 1b). Halo gravity traction (HGT) failed to improve the deformity. CT-based navigation for halo pelvic traction (HPT) construction was chosen to reduce the risk of major vessel and pelvic organ injury due to the patient’s short stature with small pelvis, and pelvic obliquity (transilium pelvic height difference of +9mm, pelvic hypoplasia of +2°, and pelvic rotation of +1.34 ratio) (Fig. 2a).

HPT construct was performed in an operating theatre connected to a Siemens Somatom AS+ 128-slice CT scanner (Fig. 2b). The patient, positioned supine, had a new halo ring with 8 pins applied, replacing the old loose pins. She was then turned prone, and two 3.5mm Schanz pins were inserted at the left iliac crest to secure the navigation tracker, with pin placement verified using inlet and outlet fluoroscopy view (Fig. 2c). CT scans were performed for navigation system calibration (Fig. 2b). Once the CT scan was complete, the machine was removed, and the surgical area was cleaned and draped. Using the CT images, the drill bit’s trajectory was navigated in coronal, sagittal, and axial views. After drilling with a 3.5mm drill bit, two 6.0 x 150mm hydroxyapatite (HA) coated Schanz pins were inserted into both posterior iliac wings at the posterior superior iliac spine (PSIS) under navigation guidance (Fig. 2d and 2e). The posterior halo pelvic frame was constructed by connecting the halo ring to the pelvic frame using two rods with extensions. The patient was then turned supine for anterior pin insertion. After drilling with a 3.5mm drill bit, two 6.0 x 200mm HA coated pins were inserted in the supra-acetabular region with a 15° angulation to the hip flexion axis to ease mobilisation, especially sitting with the frame in situ (Fig. 2f and 2g). Obturator oblique views of imaging intensifier (I/I) confirmed the placement of the supra-acetabular HA coated pins, and the anterior halo pelvic frame was constructed to complete the ring (Fig. 2h). The initial Schanz pins for the navigation tracker were then removed. The total duration of surgery was five hours.

**Fig. 3:**
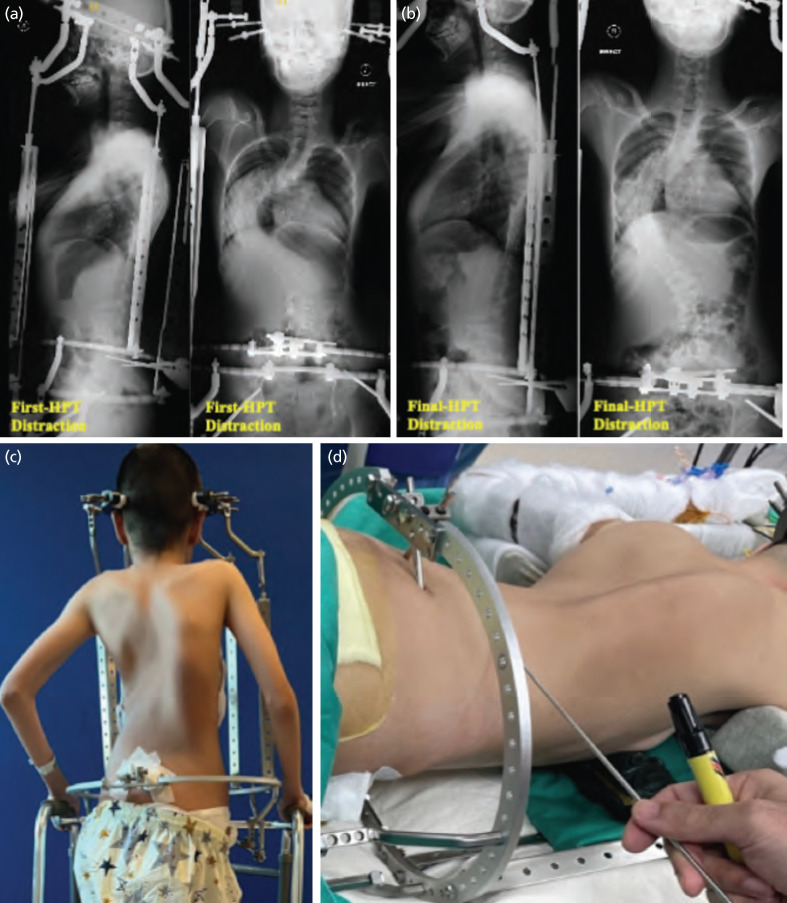
(a) Anterior-posterior and lateral views of the erect spine plain radiograph shows a Cobb angle improvement of 38° and a sagittal angle improvement of 16° with the first HPT distraction. (b) After three distractions, there was a marked improvement in the kyphotic angle (from 90° to 64°) and the Cobb angle (from 158° to 103°) with HPT. (c) Two posterior struts were shifted to the anterior for definitive spinal surgery. (d) Posterior spinal fusion done with halo pelvic frame in situ and loosened struts.

Three days post-operatively, the patient was able to sit at the bedside and started ambulation with a walking frame on the day 5. HPT distraction started two weeks later with weekly sessions for 3 weeks, achieving a total distraction of 78mm to 107mm across 4 different struts (Table I). There were no neurological complications after each distraction, confirmed by clinical neurology examinations, with no neuro-monitoring used throughout the course of distraction. Significant improvement in spinal curvature was observed, whereby the Cobb angle improved from 158° to 103° and kyphotic correction from 90° to 64° (Fig. 3a, Fig. 3b and Table I). The bilateral anterior pelvic pins complicated with mild to moderate pin site infections, which were managed with frequent dressing, bedside desloughing and antibiotics. In view of no pin loosening and sign of osteomyelitis, we kept the pins for six weeks until definitive correction surgery. Before the definitive deformity corrective surgery, two posterior struts were shifted anteriorly to facilitate the surgery done with the halo-pelvic frame in situ (Fig. 3c).

**Table I:** Amount of distraction in halo pelvic traction initiated two weeks after HPT application, along with changes in Cobb angle.

Timing of distraction	Right anterior strut	Amount of distraction (mm) Left anterior Right posterior strut strut	Left posterior strut	Cobb angle changes	Sagittal angle changes
1st distraction	45	59 35	38	38°	16°
2nd distraction	21	32 37	25	10°	6°
3rd distraction	12	16 22	24	7°	4°
Total	78	107 97	87	55°	26°

The posterior spinal fusion surgery was performed from T1 to L3 with HPT in situ. All the struts were loosened to allow further deformity correction during the surgery (Fig. 3d). No vertebral column resection was done. Post-surgery, the Cobb angle and kyphotic angle improved to 73° and 40° respectively, indicating a correction rate of 53.8% and 55.6% in the coronal and sagittal plane, respectively (Fig. 1c and 1d). The patient’s height increased from 137cm to 153cm post-operatively, and no neurological deficit were noted. A lung function test was not performed due to the patient’s inability to co-operate. She was discharged one week after the operation, with well-healed pin-site wounds. Consent for all photographs used in this case report and for the surgery was obtained from the parents after a thorough explanation of the indications, risks, and potential complications.

## Discussion

Halo-pelvic traction (HPT), developed by Hodgson, Yau, and O’Brien in the late 1960s, utilises strong distraction forces to correct various spinal deformities^3^. Despite a decline in use due to advancements in internal fixation, HPT remains crucial for pre-operative curve correction in skeletally immature patients, those with poor bone quality, pre-existing neurological deficits, or high risk of neurological injury during corrective procedures^2^. Spinal osteotomy for severe rigid scoliosis, particularly with a Cobb angle over 120°, carries risks of increased blood loss and spinal cord injury^1^. HPT significantly reduces the curve magnitude, improves curve flexibility, facilitating screw placement, besides reducing blood loss and operative time^1^.

In our patient, four weeks of weekly distraction resulted in a 34.8% improvement in coronal correction and a 28.9% improvement in sagittal correction. This pre-operative improvement allowed for definitive corrective surgery with reduced blood loss (500ml) and shorter surgical time (345 mins) for severe scoliosis^1^.

Despite its efficacy, HPT has limitations and potential complications, including pin site infection, pin loosening, extended hospital stays, and risks of hospital-acquired pneumonia. Inserting pelvic pins poses risks of major vessel, nerve injury, and intestinal perforation^2,3^. However, short-term HPT, as applied in our case, is safe since gradual traction allows for prompt detection of neurological deficits^1^.

Various approaches to constructing a pelvic frame have been reported. O'Brien’s technique involved inserting the pelvic pin in a lateral position with a through-and-through method from anterior to posterior pelvis through the iliac crest, which carries risks of intestinal perforation and major vessel injury, especially in younger patients with pelvic obliquity and muscle wasting^5^. Qi *et al* adapted HPT for patient comfort by using only an anterior pin with three pins on each side between the ilium tables^5^.

Our four-pin construct (two supra-acetabular and two posterior iliac crest pins) (Fig. 2h) with CT-based navigation reduced the risk of injury to major arteries, nerves, and abdominal/pelvic organs while providing a stable halo pelvic frame. Pelvic surgery's complexity arises from the anatomy, limited exposure, and challenges in achieving high-quality intra-operative imaging. Continuous fluoroscopy increases radiation exposure to operative staff. Factors like obesity or bowel gases impede visualisation, leading to incorrect pin placement. CT-based navigation facilitates precise pin insertion, reduces cortical perforation rates, and diminishes the risk of bowel or pelvic organ damage.

In our syndromic patient with a small, oblique pelvis, CT-based navigation enabled the placement of a supra-acetabular pin in a more lateral position (15° to the hip flexion axis) without risking cortical penetration or pelvic organ injury. This adjustment improved hip flexion and comfort during sitting and mobilisation, compared to the conventional supra-acetabular pin inserted using imaging intensifier (I/I) guidance. Posterior pin placement can cause discomfort in the supine position, but this can be mitigated by placing pillows above and below the posterior pin to relieve pressure. While CT-based navigation for pelvic pin placement aims to expedite HPT surgery, it has a steep learning curve. With more frequent application, surgery time could be further reduced.

Halo-pelvic traction remains a viable and effective option, particularly in cases of severe rigid spinal deformities to improve the curve flexibility and magnitude to facilitate the definitive corrective spinal surgery. The integration of CT-based navigation enhances pin placement precision apart from reducing HPT-related complications.
